# Impact of Drying Methods on Bioactive Compounds and Antioxidant Properties of *Kalanchoe ceratophylla*

**DOI:** 10.1155/sci5/7146758

**Published:** 2025-02-20

**Authors:** Lamhot Parulian Manalu, Himawan Adinegoro, Nenie Yustiningsih, Astuti, Rohmah Luthfiyanti, Maisaroh, Wahyu Purwanto, Subandrio, Olivia Bunga Pongtuluran, Priyo Atmaji, Taufik Hidayat, Henky Henanto, Ali Asgar, Achmad Sofian Nasori, Agus Triyono, Berna Elya, Abdullah Bin Arif

**Affiliations:** ^1^Research Center for Agroindustry, Research Organization for Agriculture and Food, National Research and Innovation Agency (BRIN), Tangerang, Indonesia; ^2^Research Center for Appropriate Technology, Research Organization for Agriculture and Food, National Research and Innovation Agency (BRIN), Tangerang, Indonesia; ^3^Phytochemistry and Pharmacognosy Laboratory, Faculty of Pharmacy, University of Indonesia, Depok, Indonesia

**Keywords:** anticancer, anticonvulsant, antidiabetic, gamma-sitosterol, herbal plant

## Abstract

*Kalanchoe* species have been used as herbal medicines in traditional Asian medicine. One of the *Kalanchoe* species that has the potential as a medicinal plant and is still limited in its studies is *Kalanchoe ceratophylla*. This study investigated the effects of drying methods, namely, freeze drying (FD) and hot air drying (HAD), on bioactive compounds, phenolics, flavonoids, and antioxidants in *Kalanchoe ceratophylla* leaves and stems. The content of bioactive compounds in *K*. *ceratophylla* leaves and stems was identified with gas chromatography-mass spectrometry (GC-MS) analysis. In addition, the antioxidant activity of *K. ceratophylla* was also measured. The results of GC-MS on *K. ceratophylla* contain major bioactive compounds including gamma.-Sitosterol, Glutinol, Friedelan-3-one, Squalene, Ergost-5-en-3-ol, (3.β.)-, Erythritol, and Neophytadiene. The main component identified in the leaf and stem extracts of *K. ceratophylla* is gamma-sitosterol (± 15%), which shows anticancer and antidiabetic effects. The antioxidant activity in *K. ceratophylla* with the FD method was higher than that with the HAD method. This study suggests that FD was considered appropriate and should be used to maintain the content of important bioactive compounds and antioxidant activity in *K. ceratophylla*. These findings suggest that further research and development of bioactive compounds essential for their pharmacological properties in *K. ceratophylla* may be warranted, with the potential for developing new drugs.

## 1. Introduction

Recently, people in developing countries such as Indonesia have become more concerned and aware of the use of herbal medicines to cure various diseases. One of the raw materials for herbal medicines comes from plants, commonly called herbal plants. Various herbal plants have been widely used as healing agents for various human diseases [1]. In addition, it is estimated that about a quarter of the medicines used by humans come from plants [2, 3]. Generally, herbal plants contain high antioxidants [4, 5]. Plants contain secondary metabolites that function as sources of antioxidants, such as phenolic compounds and flavonoids [6]. Medicinal plants are also rich in secondary metabolites that function as antioxidants, anti-inflammatories, anticancer, antiviral, antifungal, and antibacterial [7, 8]. Antioxidants are chemical compounds that can reduce free radicals and the rate of production and lipid peroxidation in the human body, which causes various human diseases and aging [9]. Antioxidants function as free radical scavengers and treat various diseases that produce reactive oxygen species (ROS) and damage cells, whereas antioxidants convert oxidants into less harmful forms [10]. Due to their antioxidant properties, herbal medicines use plants as natural components [5]. The phytochemicals in medicinal plants, considered bioactive compounds, have been confirmed to be safe, effective, relatively inexpensive, and recently predicted as a suitable substitute for antibiotics [11]. One of the herbal plants that have the potential as an herbal medicine is the *Kalanchoe* species.

*Kalanchoe* species have been used as herbal medicines in traditional Asian medicine, namely, *K. crenata* (Andrews) Haw., *K. integra* (Medik.) Kuntze, *K. laciniata* (L.) DC., and *K. pinnata* (Lam.) Pers. [12, 13]. In addition, *K. crenata* (Andrews) Haw. and *K. pinnata* have been traditionally used by Brazilians to treat abscesses, burns, inflammation, rheumatism, and wounds [14]. In Indonesia, fresh leaves of *K. pinnata* and *K. prolifera* are commonly applied externally as a poultice for wounds, burns, blisters, and fever [15, 16]. Furthermore, several biological activities have been shown from *Kalanchoe* sp. plant extraction, including anti-inflammatory, antimicrobial, wound healing, muscle relaxant, and antitumor activities [17–20]. One of the *Kalanchoe* species that has the potential as a medicinal plant and is still limited in its studies is *K. ceratophylla*. As a medicinal plant, *K. ceratophylla* is vulnerable to extinction. Therefore, Vuorela et al. [21] stated that it is necessary to identify the content of bioactive compounds in medicinal plants for scientific validation or discovery of the main compounds to be used as herbal medicines.

No pharmacological study has been conducted to identify the potential of *K. ceratophylla* phytochemical compounds as herbal plants for traditional medicine. As a plant with high water content, *Kalanchoe ceratophylla* is easily damaged by microorganisms and enzyme activity during storage. Therefore, *K. ceratophylla* must go through a preservation process so that it can be utilized during storage. Drying is one of the most widely used methods in preserving agricultural products [22]. Heat drying is the most popular conventional method because of its easy application [23, 24]. However, heat drying can cause adverse effects, such as deleting critical bioactive compounds [24]. On the other hand, freeze drying (FD) is a low-temperature dehydration technique based on the sublimation of water present in a product, which reduces water activity [25]. The FD produces quality products with minimal degradation of bioactive compounds [26].

The current study will analyze bioactive compounds in *K. ceratophylla* extract at different drying using gas chromatography-mass spectrometry (GC-MS). The GC-MS method is one of the methods that can be used to identify bioactive compounds in plants. The GC-MS method has been proven to identify several bioactive compounds and evaluate various phytoconstituents in plants used in cosmetics, medicines, pharmaceutical or food industries, and environmental and forensic applications [2]. Therefore, the study aims to identify bioactive compounds and antioxidant activity in *K. ceratophylla* extracts with two different drying methods.

## 2. Materials and Methods

### 2.1. Plant Material

Mature whole plants of *K. ceratophylla* were collected from farmlands and gardens in the Depok District of West Java Province, Indonesia. The location is situated at a longitude of 106.77° E and a latitude of 6.24° S. The harvesting period for *K. ceratophylla* in this research was during June and July, with the plants harvested four months after planting. The plants were identified at the Herbarium Bandungense (FIPIA), which is located at the School of Life Sciences and Technology, Institut Teknologi Bandung (ITB), as well as at the Laboratory of Gedung Utara, Bogor Botanical Gardens, Indonesia.

### 2.2. Drying Procedure

The leaves and stems of *K. ceratophylla* were separated manually. These samples were dried using two different methods: FD and hot air drying (HAD). The leaves and stems were frozen in a freezer for 12 h for the FD method. After freezing, the material was dried in a freeze dryer at −55°C for 24 h. In contrast, in the drying process using the HAD method, the leaves and stems of *K. ceratophylla* were dried at 45°C for 48 h. After drying, the samples from both methods were ground into a powder and stored at approximately 4°C.

### 2.3. Extraction Procedure

A total of 1 g of powder from the leaves and stems of *K. ceratophylla* was extracted using both FD and HAD methods with 100 mL of methanol 99.8% (Merck, catalog number 1.06009.2500). The mixture was then macerated for 24 h at room temperature. After maceration, the solution was filtered through Whatman filter paper number 41, yielding approximately 100 mL of supernatant with a concentration of 10,000 ppm. The supernatant was stored as a stock solution at −20°C until further analysis.

### 2.4. Determination of Total Phenolic Content (TPC)

The TPC of *K. ceratophylla* was determined using the Folin–Ciocalteu (Merck, catalog no 1.09001.0100) reagent method, as described by Arif et al. [27], with gallic acid (Merck, catalog no 8.42649.0250) as the standard phenolic compound. The TPC analysis involved mixing 50 μL of *K. ceratophylla* leaf and stem extract solutions (applied via FD and HAD methods) in a test tube with 1 mL of the Folin–Ciocalteu reagent, which had been diluted 1:4 with distilled water. After mixing thoroughly, 1 mL of 10% sodium carbonate (Na_2_CO_3_) was added. The mixture was allowed to stand in the dark for one hour. Absorbance was then measured spectrophotometrically using a Hitachi instrument (document number U1800) at a wavelength of 750 nm. The total concentration of phenolic compounds in the leaf and stem extracts was determined using the standard gallic acid graph equation, with gallic acid concentration plotted on the *X*-axis and the corresponding absorbance on the *Y*-axis. The results were expressed as milligrams of gallic acid equivalents (GAE) per gram of dry weight.

### 2.5. Determination of Total Flavonoid Content (TFC)

The TFC in the extract of *K. ceratophylla* was determined using the aluminum chloride colorimetric method as described by Sarker and Oba [28]. In this procedure, 500 μL of the *K. ceratophylla* extract was transferred to a test tube, followed by the addition of 1.5 mL of methanol, 0.1 mL of 10% aluminum chloride (Merck, catalog no. 1.01084.1000), 0.1 mL of 1 M potassium acetate (Merck, catalog no. 104820), and 2.8 mL of distilled water. The mixture was allowed to stand at room temperature for 30 min. Afterward, the absorbance of the reaction mixture was measured spectrophotometrically using a Hitachi U1800 instrument (Hitachi, Tokyo, Japan). Quercetin (Merck, catalog no. Q4951) was used as a standard compound, and TFC was expressed as μg quercetin equivalents (QE)/g dry weight.

### 2.6. GC-MS Analysis

The analysis of bioactive compounds was conducted using GC-MS with an Agilent Technologies Type 7890B system, employing methanol as the solvent. The detection utilized an electron ionization system with an ionization energy of 70 eV. Helium gas was used as the carrier gas at a constant flow rate of 1 mL/min. To prepare the sample, 1–1.5 mL of the supernatant from the stock solution was filtered through a 0.22 μm syringe filter. The filtered sample was then placed into a vial and injected into the GC-MS. The quantification of the identified components was investigated using the percent relative peak area [29].

### 2.7. Antioxidant Activity

Antioxidant activity was measured using the diphenyl-picryl-hydroxyl (DPPH-TCI Japan) radical degradation method described by Ahmed et al. [11]. This method is simple and quick, based on the principle of quantitatively measuring antioxidant activity. A total of 10 μL of the leaf and stem extract of *K. ceratophylla* solution was added to a test tube containing 4 mL of distilled water and 1 mL of 250 μM DPPH solution. The mixture was thoroughly mixed and allowed to stand for 30 min in the dark. The absorbance was then measured at 517 nm using a Hitachi spectrophotometer (Hitachi, Tokyo, Japan). Negative controls were prepared in the same manner, using absolute methanol, while a 0.1% (w/v) ascorbic acid solution in methanol was used as a positive control at concentrations ranging from 0 to 80 μg/mL. The antioxidant activity was estimated based on the percentage of DPPH radicals that were scavenged, calculated using the following formula:

(1) $$\begin{array}{c} \text{DPPH radical scavenging activity }\left(\%\right)=\left[\frac{\left(A0-\text{As}\right)}{A0}\right]\times 100. \end{array}$$

*A*0 is the absorbance of the negative control. As is the absorbance of the sample.

The IC (inhibition concentration) value indicates how much a compound suppresses free radicals. Specifically, the IC_50_ value is defined as the concentration of the test compound required to reduce free radicals by 50%. To determine the IC_50_ value, plot the extract concentration (*X*) against the DPPH inhibition percentage (*Y*) and perform a linear regression analysis. The IC_50_ can be estimated from the fitted line using the equation *Y* = *aX* + *b*, where IC_50_ = (50 − *b*)/*a*.

### 2.8. Statistical Analysis

Data were analyzed with a one-way analysis of variance. The significant differences among the treatment means were determined by least significant difference (LSD) at a probability level of 5%. Statistical analyses of data were performed by SAS Portable Version 9.1.3. The data were reported as mean ± standard error (SE) of three replications. Graphs were plotted using Excel 2016.

## 3. Result

### 3.1. TPC and TFC in *K. ceratophylla* Extracts

The TPC and TFC in *K. ceratophylla* extract with the FD method were higher than those with HAD in the leaves and stems of *K. ceratophylla* (Table 1). The TPC in the *K. ceratophylla* leaf extract with the FD method was 4.25 mg GAE/g, which was 1.17 mg GAE/g higher than the HAD method (Table 1). Likewise, in the *K. ceratophylla* stem extract, the TPC content with the FD method was 0.51 mg GAE/g higher than the HAD method (Table 1). Furthermore, the TFC in the *K. ceratophylla* leaves and stems extract using the FD method was found to be higher than that obtained with the HAD method (Table 1).

### 3.2. GC-MS Composition of *Kalanchoe ceratophylla* Extract

**Figure 1:**
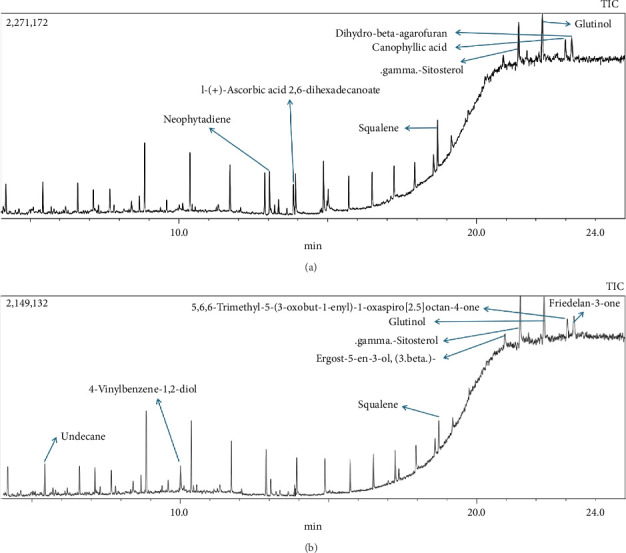
GC-MS chromatogram of methanol extract of *Kalanchoe ceratophylla* leaves with (a) hot air drying (HAD) and (b) freeze drying (FD).

**Figure 2:**
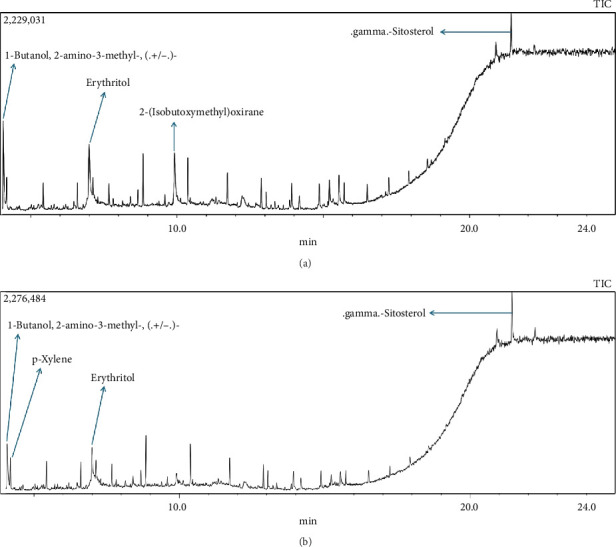
GC-MS chromatogram of methanol extract of *Kalanchoe ceratophylla* stem with (a) hot air drying (HAD) and (b) freeze drying (FD).

**Figure 3:**
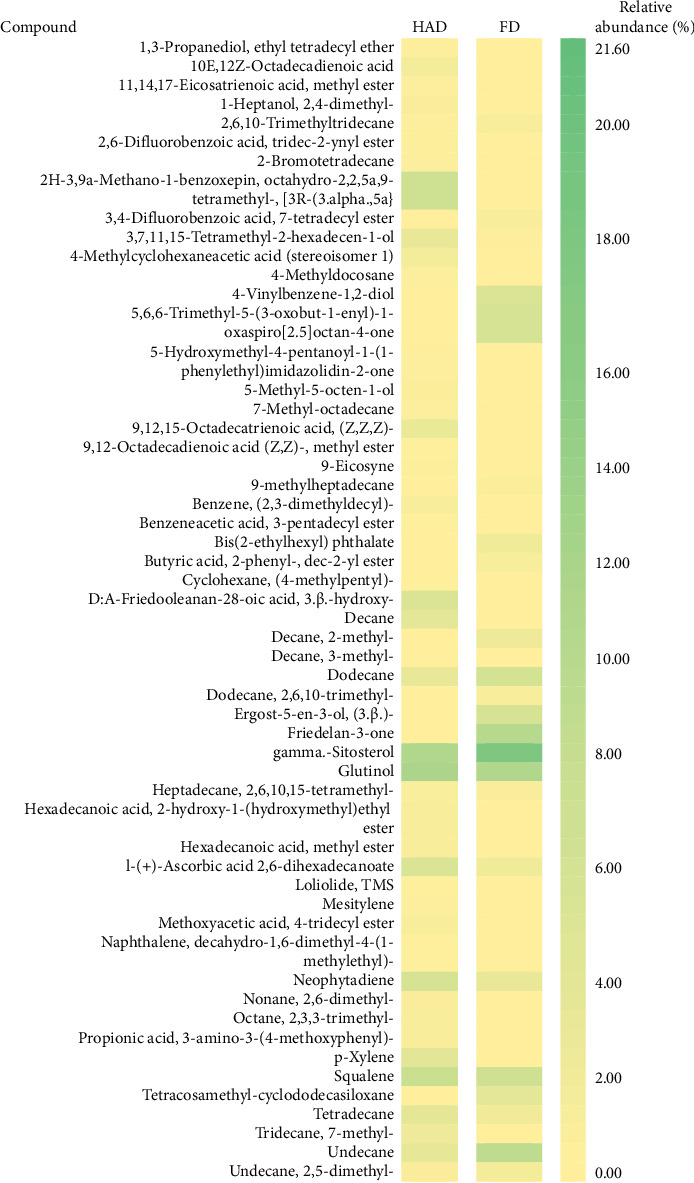
Heatmap of bioactive compounds in *Kalanchoe ceratophylla* leaves with different drying.

**Figure 4:**
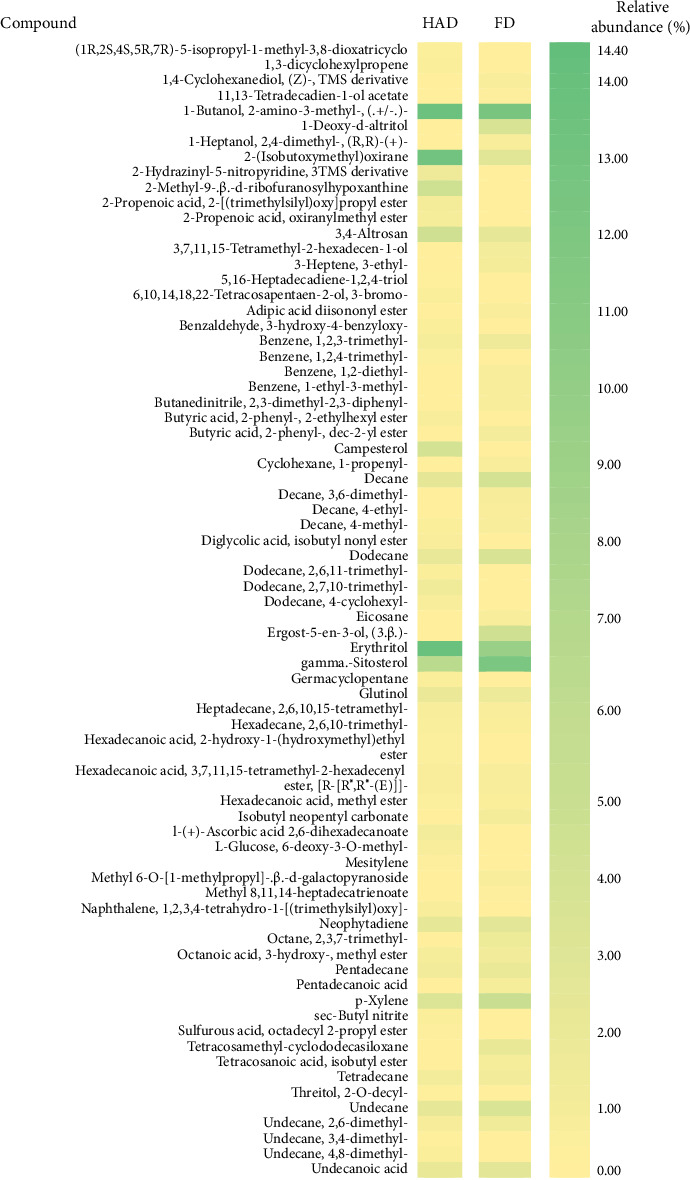
Heatmap of bioactive compounds in *Kalanchoe ceratophylla* stem with different drying.

In this study, methanol extracts of leaves and stems of *K. ceratophylla* were used for GC-MS analysis. GC-MS results revealed about 55 and 72 bioactive compounds in the leaves and stems of *K. ceratophylla*, respectively. These compounds are presented with molecular formulas and percentages of the area (Figures 1, 2, 3, and 4 and Table 2). There are major bioactive compounds that are important and beneficial for health contained in the extracts of leaves and stems of *K. ceratophylla*, including Ergost-5-en-3-ol, (3.β.)-, Friedelan-3-one, gamma.-Sitosterol, Glutinol, Neophytadiene, p-Xylene, Squalene, Campesterol, and Erythritol. The drying method affects the content of major bioactive compounds in the leaves and stems of *K. ceratophylla*. The content of Ergost-5-en-3-ol, (3.β.)-, Friedelan-3-one, gamma.-Sitosterol, and Squalene in *K. ceratophylla* leaf extract with the FD method was higher than that with HAD. Conversely, the content of Neophytadiene and p-Xylene in *K. ceratophylla* leaf extract with the FD method was lower than that with HAD (Figure 3). Furthermore, in the *K. ceratophylla* stem extract, Ergost-5-en-3-ol, (3.β.)-gamma-Sitosterol, and p-Xylene contents obtained using the FD method were higher than those obtained through the HAD method. On the other hand, the content of Erythritol and Campesterol in *K. ceratophylla* stem extract with the FD method was lower than that with HAD (Figure 4).

In the extract of *K. ceratophylla* leaves using the FD method, the GC-MS results showed that the compound gamma.-Sitosterol had the highest concentration (17.79%), followed by Glutinol (10.84%), Friedelan-3-one (10.01%), Undecane (9.06%), Squalene (6.65%), and Ergost-5-en-3-ol, (3.β.)- (5.69%) (Figure 3). Meanwhile, in the extract of *K. ceratophylla* leaves with the HAD method, the GC-MS results showed that the Glutinol compound was found at the highest concentration (11.51%), followed by gamma.-Sitosterol (10.85%), Squalene (7.21%), 2H-3,9a-Methano-1-benzoxepin, octahydro-2,2,5a,9-tetramethyl-, [3R-(3.alpha.,5a} (6.87%), D:A-Friedooleanan-28-oic acid, 3.β.-hydroxy- (5.25%), and Neophytadiene (5.7%) (Figure 3).

The GC-MS analysis of *K. ceratophylla* extracts revealed distinct differences in the bioactive compound content between the leaves and stems, depending on the extraction method used. For the *K. ceratophylla* stem extract obtained using the FD method, the compound 1-Butanol, 2-amino-3-methyl- (±) was found to have the highest concentration at 12.46%. This was closely followed by gamma-Sitosterol at 12.24%, Erythritol at 9.44%, and Neophytadiene at 2.76% (Figure 4). In contrast, the *K. ceratophylla* stem extract using the HAD method showed that Erythritol had the highest concentration at 13.84%. This was followed by 1-Butanol, 2-amino-3-methyl- (±) at 13.69%, 2-(Isobutoxymethyl)oxirane at 13.05%, gamma-Sitosterol at 6.39%, and Campesterol at 3.99% (Figure 4).

### 3.3. Evaluation of Antioxidant Potentials of Extracts of *K. ceratophylla*

Differences in drying methods affect the antioxidant activity of *K. ceratophylla* extract (Table 3). The antioxidant activity (IC_50_) of *K. ceratophylla* leaf extract with the FD method was approximately 2-fold higher than that with the HAD method (Table 3). In addition, the antioxidant activity (IC_50_) of *K. ceratophylla* stem extract with the FD method was higher than HAD by 26.17 μg/mL (Table 3).

## 4. Discussion

Bioactive compounds, particularly phenolics and flavonoids found in plants, significantly promote human health [46]. As common polyphenolic compounds, phenols and flavonoids are vital bioactive constituents that act as natural antioxidants to benefit human well-being [47]. Previous study by Nascimento et al. [20] highlighted that flavonoids are likely *Kalanchoe* species' most recognized and potent molecules. Moreover, Costa et al. [17] emphasized that flavonoids constitute the *Kalanchoe* species' most important chemical group, considering the volume of reports and their biological activities. In this study, the flavonoid and phenolic content in the leaf and stem extracts of *K. ceratophylla* was high. Moreover, the HAD method revealed that the TPC in the leaves and stems of *K. ceratophylla* was lower than that of the FD method. This suggests that heat treatment can lead to a reduction in phenolic compounds. Kayacan et al. [48] noted that heat treatment makes phenolic compounds more susceptible to oxidation. During the conventional drying process utilizing hot air, phenolic compounds are degraded [49]. In contrast, the FD method preserves the TPC in *K. ceratophylla* extract. The very low drying temperatures employed in the FD method effectively inhibit phenolic degradation, thereby maintaining the TPC within the sample [26]. The FD method is regarded as the optimal drying technique for preserving bioactive compounds and other nutrients found in food [50]. Furthermore, this study demonstrated that the HAD method yielded lower TFC in *K. ceratophylla* extract than the FD method. HAD negatively impacts flavonoid content [51–53]. The reduction in TFC is attributed to the harsh drying conditions, which can disrupt phytochemicals and lead to the degradation of certain flavonoids [51]. Conversely, Mullen et al. [54] reported that the freezing process did not significantly affect the TFC in raspberries. Therefore, the FD method is deemed appropriate for maintaining phenolic and flavonoid content when drying *K. ceratophylla* as an herbal medicine. Additionally, given the recognized benefits of phenolics and flavonoids for human health, these findings bolster the case for utilizing *K. ceratophylla* in traditional medicine.

*K. ceratophylla* is an herbal plant rich in important bioactive compounds and is commonly found in Indonesia. This study utilized GC-MS to identify the bioactive compounds present in *K. ceratophylla*. There are essential bioactive compounds contained in *K. ceratophylla* extract, including gamma.-sitosterol, Glutinol, Friedelan-3-one, Squalene, Ergost-5-en-3-ol, (3.β.)-, Erythritol, and Neophytadiene.

Gamma-Sitosterol is a bioactive compound from herbal plants used for traditional medicine in human health. Currently, γ-sitosterol has been reported as a traditional medicine, especially as an antidiabetic, because it has antihyperglycemic activity by increasing insulin secretion in response to glucose [35, 36, 55]. In addition, Sundarraj et al. [37] also reported that γ-sitosterol also functions as an anticancer through growth inhibition and cell cycle arrest in cancer cell apoptosis. Furthermore, Endrini et al. [56] also reported that γ-sitosterol is cytotoxic to colon and liver cancer cell lines. In this study, the relative abundance of γ-sitosterol in *K. ceratophylla* extract was high (10%–18%), but the γ-sitosterol content with the FD method was higher than that with the HAD method. Bioactive compounds, including sterols, are easily degraded when exposed to hot air. Therefore, the FD method is considered appropriate for maintaining the content of γ-sitosterol in the leaves and stems of *K. ceratophylla*.

*K. ceratophylla* is a potential herbal plant with anticancer properties [17, 18, 20]. In this study, *K. ceratophylla* contains Glutinol. Previous studies have indicated that Glutinol with the chemical formula C_30_H_50_O has anti-inflammatory, anticancer, and antidiabetic properties [38, 40, 41]. As a triterpenoid compound, Glutinol has been shown to inhibit the growth of human cancer cells [39]. In this study, the Glutinol content was higher in the leaf extract than in the stem extract. Furthermore, the drying methods did not impact the Glutinol content in *K. ceratophylla*. The relative abundance of Glutinol in the leaf extract was observed to be 10%–12%. Therefore, *K. ceratophylla* demonstrates significant potential as an herbal medicine for anticancer.

In addition to Glutinol, Squalene is an important triterpene that plays a crucial role in human health. Squalene is a natural and essential component that acts as an anticancer agent within the human body due to its chemopreventive properties [30, 57, 58]. In this study, Squalene was exclusively found in the leaf extract of *K. ceratophylla*, and the method of drying did not influence the Squalene content in this plant.

Friedelan-3-one has been studied for its potential biological activities and medicinal properties. This compound is a pentacyclic triterpene isolated from various plant species. Ee et al. [59] reported that Friedelan-3-one inhibits the growth of MBA-MD-231 breast cancer cells, which are human estrogen receptor negative. As an anticancer agent, Friedelan-3-one demonstrates antiproliferative properties against various cancer cell lines [32]. Additionally, Friedelan-3-one has been reported to possess anticonvulsant and antiulcer activities [33, 34]. In this study, Friedelan-3-one was exclusively found in the leaf extract of *K. ceratophylla*. The HAD method was shown to reduce the content of Friedelan-3-one in *K. ceratophylla*, while the FD method effectively prevents its degradation. Therefore, the FD method is considered more suitable for maintaining Friedelan-3-one levels in *K. ceratophylla* leaves, which is beneficial for its anticancer and anticonvulsant properties.

Another bioactive compound that has anti-cancer properties is Ergost-5-en-3-ol (3.β.). This phytosterol has been shown to inhibit the growth of cancer cells [30, 31]. In this study, the Ergost-5-en-3-ol (3β) compound was found in the leaf and stem extracts of *K. ceratophylla*. However, HAD treatment degraded the content of these compounds. Bioactive compounds, including the sterol group, are easily degraded and damaged when exposed to hot air. Therefore, the leaf and stem extracts of *K. ceratophylla* through the FD process can be used as alternative medicinal ingredients for anticancer.

Other bioactive compounds that function as anticonvulsants include Neophytadiene. Neophytadiene is an anticonvulsant with anxiolytic-like activity [42]. Neophytadiene has analgesic, antipyretic, anti-inflammatory, and antioxidant properties [60]. Neophytadiene, a diterpene that can interact with GABA receptors, produces anxiolytic, antidepressant, anticonvulsant, and sedative effects [42]. In this study, Neophytadiene was found in leaf and stem extracts of *K. ceratophylla*. In addition, the drying method did not affect the Neophytadiene content in *K. ceratophylla*. Therefore, *K. ceratophylla* can be used as a potential herbal plant for anticonvulsants.

In addition to containing bioactive compounds that function as anticancer and anticonvulsants, *K. ceratophylla* also contains Erythritol compounds that can be used as food sweeteners. Erythritol is a low-calorie sweetener, so it is safe for people with diabetes [44, 61]. In this study, Erythritol was only found in *K. ceratophylla* stem extract.

This study used the DPPH method to calculate the antioxidant activity in *K. ceratophylla*. The findings indicated that the antioxidant activity (IC_50_) of *K. ceratophylla* ranged from 25 to 51 μg/mL. In previous research, Bogucka-Kocka et al. [62] reported IC_50_ values of 61.5 μg/mL for *K. milloti* and 90.6 μg/mL for *K. pinnata*. The results also reported that the antioxidant activity (IC_50_) of *K. ceratophylla* obtained through the FD method was greater than that achieved with the HAD method. The HAD process tends to diminish the product's antioxidant properties due to the degradation of bioactive compounds caused by higher thermal exposure. As the thermal load increases, the antioxidant activity decreases. Consequently, the FD method is considered more effective for preserving the antioxidant activity in *K. ceratophylla*.

## 5. Conclusion

*Kalanchoe ceratophylla* is a valuable source of natural compounds that have the potential as herbal plants which function as anticancer, antidiabetic, and anticonvulsant. The results of GC-MS on *K. ceratophylla* contain major bioactive compounds including gamma.-Sitosterol, Glutinol, Friedelan-3-one, Squalene, Ergost-5-en-3-ol, (3.β.)-, Erythritol, and Neophytadiene. The main component identified in the leaf and stem extracts of *K. ceratophylla* is gamma-sitosterol (± 15%), which shows anticancer and antidiabetic effects. The FD method is considered appropriate and can be used to maintain the content of important bioactive compounds and antioxidant activity in *K. ceratophylla*. These findings suggest that further research and development regarding the bioactive compounds in *K. ceratophylla* may be beneficial, potentially leading to the creation of new pharmaceuticals.

## Figures and Tables

**Table 1:** Total phenolic and total flavonoid content in *K. ceratophylla*.

Organ of *K. ceratophylla*	Parameters	Drying method	
		HAD	FD
Leaves	Total phenolic content (mg GAE/g)	3.08 ± 0.23^b^	4.25 ± 0.25^a^
	Total flavonoid content (μg QE/g)	10.04 ± 1.12^b^	12.27 ± 0.51^a^

Stem	Total phenolic content (mg GAE/g)	3.27 ± 0.25^b^	3.78 ± 0.17^a^
	Total flavonoid content (μg QE/g)	2.47 ± 0.32^b^	3.49 ± 0.44^a^

*Note:* Different letters in the same lines indicate the significant difference by the LSD test.

Abbreviations: FD = freeze drying; HAD = hot air drying.

**Table 2:** Major bioactive compounds identified in the methanol extract of *K. ceratophylla*.

No.	Compound	Molecular formula	Chemical structure	Bioactivity	Reference
1	Ergost-5-en-3-ol, (3.β.)-	C_28_H_48_O	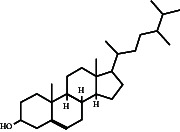	Anticancer	[30, 31]

2	Friedelan-3-one	C_30_H_50_O	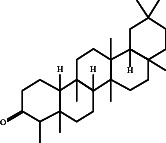	Anticancer, anticonvulsant	[32–34]

3	gamma.-Sitosterol	C_29_H_50_O	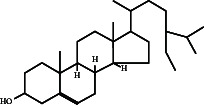	Antidiabetic, anticancer	[35–37]

4	Glutinol	C_30_H_50_O	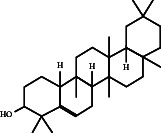	Anti-inflammatory activity, anticancer, and antidiabetic	[38–41]

5	Neophytadiene	C_20_H_38_	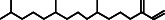	Antiproliferative, anxiolytic, anticonvulsant, antimicrobial, antifungal, anti-inflammatory, and antioxidant	[30, 42, 43]

6	Squalene	C_30_H_50_	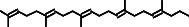	Antioxidant, anticancer	[30]

7	Erythritol	C_4_H_10_O_4_	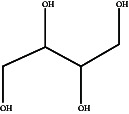	Noncaloric, noncariogenic, nonglycemic, and antioxidant	[44, 45]

**Table 3:** Antioxidant activity (IC_50_) of *K. ceratophylla*.

Organ of *K. ceratophylla*	IC_50_ (μg/mL)	
	HAD	FD
Leaves	26.27 ± 2.21^b^	49.24 ± 3.35^a^
Stem	24.76 ± 2.43^b^	50.93 ± 5.21^a^

*Note:* Different letters in the same lines indicate the significant difference by the LSD test.

Abbreviations: FD = freeze drying; HAD = hot air drying.

## Data Availability

The data used to support the findings of the study are included within the article.
